# A Bayesian Poisson-lognormal Model for Count Data for Multiple-Trait Multiple-Environment Genomic-Enabled Prediction

**DOI:** 10.1534/g3.117.039974

**Published:** 2017-03-29

**Authors:** Osval A. Montesinos-López, Abelardo Montesinos-López, José Crossa, Fernando H. Toledo, José C. Montesinos-López, Pawan Singh, Philomin Juliana, Josafhat Salinas-Ruiz

**Affiliations:** * Facultad de Telemática, Universidad de Colima, 28040, México; † Departamento de Matemáticas, Centro Universitario de Ciencias Exactas e Ingenierías (CUCEI), Universidad de Guadalajara, 44430 Jalisco, México; ‡ International Maize and Wheat Improvement Center (CIMMYT), 06600 México, D.F., México; § Departamento de Estadística, Centro de Investigación en Matemáticas (CIMAT), Guanajuato 36240, México; ** Colegio de Postgraduados, Campus Córdoba, Km. 348 Carretera Federal Córdoba-Veracruz, Amatlán de los Reyes, 94946, México

**Keywords:** count phenotype, multi-trait, multi-environment, Bayesian, genomic-enabled prediction, GenPred, shared data resource, genomic selection

## Abstract

When a plant scientist wishes to make genomic-enabled predictions of multiple traits measured in multiple individuals in multiple environments, the most common strategy for performing the analysis is to use a single trait at a time taking into account genotype × environment interaction (G × E), because there is a lack of comprehensive models that simultaneously take into account the correlated counting traits and G × E. For this reason, in this study we propose a multiple-trait and multiple-environment model for count data. The proposed model was developed under the Bayesian paradigm for which we developed a Markov Chain Monte Carlo (MCMC) with noninformative priors. This allows obtaining all required full conditional distributions of the parameters leading to an exact Gibbs sampler for the posterior distribution. Our model was tested with simulated data and a real data set. Results show that the proposed multi-trait, multi-environment model is an attractive alternative for modeling multiple count traits measured in multiple environments.

Plant breeders need more efficient models for performing genomic selection for multiple-traits and multiple-environments for count data. Count data are those dependent variables that take values 0, 1, 2,… without an explicit upper limit. These types of dependent variables are common in genomic selection, for example: panicle number per plant, seed number per plant, number of infected spikelets per plant, *etc*. Due to its simplicity and its ability to generate samples from high-dimensional probability distributions, the Gibbs sampler is one of the most popular computationally intensive methods for fitting complex multilevel models (Park and van Dyk 2009). This method is also very popular for modeling normal and binary responses when efficient closed-form Gibbs samplers have been developed. However, obtaining a closed-form Gibbs sampler for count data is not straightforward. For this reason, Montesinos-López *et al.* (2015, 2016a) in the context of genomic-enabled prediction and genomic selection proposed closed-form Gibbs samplers for multilevel models for univariate count responses with and without the genotype × environment interaction (G × E) term that helps fill the lack of closed-form Gibbs samplers for count data. Although these models are helpful for modeling univariate count responses, many times breeders record phenotypic data for multiple counts. Scientists must take advantage of correlated traits to improve the prediction of unobserved genotypes and to increase the prediction accuracy of other count traits that are difficult to measure but that are associated with traits that are easy to measure. The available univariate count models are not appropriate for dealing with these situations.

Since prediction problems are ubiquitous and of great interest and importance in statistical science, more attention has been given to parametric inference than to predictive inference (Harville 2014). However, thanks to the efforts of scientists like C.R. Henderson, currently there is a lot of evidence that the model-based approach to prediction is a useful tool for predicting future observations, and many linear mixed effects models have been developed for predicting future observations (Harville 2014). Most of these models are valid for normal responses, but few have been developed for discrete outcomes and very few for predicting more than one trait at a time. Thus there is a great need for developing more univariate and multivariate models for non-normal traits. Developing these models is very challenging since in predictive inference, inferences are sought about tangible quantities that are regarded as unobservable at the time of the inferences; the focus is on quantities that will, or could, become observable in the future, or that can be observed at the time of the inferences but only with an unacceptable delay or with undue effort or expense (Harville 2014). These quantities are regarded as the realizations of the elements of an unobservable random vector, say an M-dimensional unobservable random column vector **w**, and/or as realizations of linear combinations (or other functions) of the elements of **w** (Harville 2014).

Poisson and negative binomial (NB) distributions are the most common random variables used for modeling count data; NB distribution is preferred when there is evidence of considerable overdispersion. In the Bayesian context, estimating the overdispersion parameter in the NB distribution is challenging because Metropolis-Hastings algorithms are used most of the time; this is computationally expensive and not practical for use in genomic-enabled prediction where data sets are large. Montesinos-López *et al.* (2015, 2016a) proposed a Gibbs sampler for NB distribution but it is not computationally efficient. Other authors have proposed using the Poisson-lognormal distribution to model count data to account for overdispersion (Williams and Ebel 2012). The Poisson component of the Poisson-lognormal distribution accommodates integer inputs (or outputs) to describe the actual number of counts observed within a single unit or sample, while the lognormal component of the distribution describes the overdispersion in the Poisson rate parameter due to clustering of some factors and describes how the average of these factors varies across the population (Williams and Ebel 2012). Adding this lognormal component to the predictor of a Poisson model is very helpful for accommodating a general correlation structure between traits when more than one trait is under study (Ma *et al.* 2008).

Based on the previous considerations, the main goal of this research is to extend the genomic-enabled Bayesian prediction model for count data with genotype × environment (G × E) interaction to the context of multiple traits under a Poisson-lognormal model. Since nowadays scientists measure multiple count traits in multiple environments, the joint modeling of multiple count traits can help to increase prediction accuracy and parameter estimation accuracy, and reduce trait selection bias. This argument is very well documented for continuous phenotypes; see, for example, Henderson and Quaas (1976), Pollak *et al.* (1984), Schaeffer (1984), Jiang *et al.* (2015), and Montesinos-López *et al.* (2016b). It is reasonable that it could also substantially help in the context of multivariate count phenotypes.

## Materials and Methods

### Experimental data

First we describe real phenotypic and genotypic experimental data used to illustrate the results of the new Poisson-lognormal model. Then we explain the theory (joint posterior density and prior specification) of the proposed model, the Gibb sampler, and its implementation. We also describe how to assess prediction accuracy and simulated data sets, genomic-enabled prediction models, and give the link to the data and software availability.

### Phenotypic data

The phenotypic data set used included 182 spring wheat lines developed by the International Maize and Wheat Improvement Center (CIMMYT) that were assembled and evaluated for resistance to *Fusarium graminearum* at El Batan experiment station in Mexico in 2011 in three experiments. For the application, we call these three experiments Env1, Env2, and Env3. In all the experiments (environments), the genotypes were arranged in a randomized complete block design, in which each plot comprised two 1-m double rows separated by a 0.25-m space. *Fusarium* head blight (FHB) severity data were collected 20 and 30 d before maturity by counting symptomatic spikelets on five randomly selected spikes in each plot. We used the counts collected at 20 d as trait 1 and the counts collected at 30 d as trait 2. These data sets were taken from the data used by Montesinos-López *et al.* (2016a) in their paper for count data with genotype × environment interaction.

### Genotypic data

DNA samples were extracted from young leaves 2–3 wk old taken from each line, using Wizard Genomic DNA purification (Promega) following the manufacturer’s protocol. DNA samples were genotyped using an Illumina 9K SNP chip with 8632 single nucleotide polymorphisms (SNPs) (Cavanagh *et al.* 2013). For a given marker, the genotype for the ith line was coded as the number of copies of a designated marker-specific allele carried by the ith line (absence = zero and presence = one). SNP markers with unexpected AB (heterozygous) genotype were recoded as either AA or BB based on the graphical interface visualization tool of GenomeStudio (Illumina) software. SNP markers that did not show clear clustering patterns were excluded. In addition, 66 simple sequence repeat markers were screened. After filtering the markers for 0.05 minor allele frequency and deleting markers with >10% of no calls, the final set of SNPs included 1635 SNPs.

### Statistical model

Let $Y_{ijk}^{\left(l\right)}$ be the phenotypic response of trait l, in replication k, in environment i for genotype j. Conditionally on $b_{j}^{\left(l\right)},$ $b_{ij}^{\left(l\right)},$ and $c_{ijk}^{\left(l\right)},$ we assume that the distribution of $Y_{ijk}^{\left(l\right)}$ is a Poisson distribution with mean equal to $\mu_{ijk}^{\left(l\right)}=\exp\left(\eta_{ijk}^{\left(l\right)}\right),$ with the following linear predictor:

$$\eta_{ijk}^{\left(l\right)}=\beta_{i\;}^{\left(l\right)}+b_{j}^{\left(l\right)}+b_{ij}^{\left(l\right)}+c_{ijk}^{\left(l\right)}$$ (1)

where $\beta_{i\;}^{\left(l\right)}$ is the intercept of environment i in trait l, $b_{j}^{\left(l\right)}$ is the random effect of line j in trait l, $b_{ij}^{\left(l\right)}$ is the interaction random effect of environment i, line j and trait l, and $c_{ijk}^{\left(l\right)}$ is an individual random effect for the response in the replication k, environment i, line j, and trait l; the $c_{ijk}$ are jointly distributed as $c_{ijk}=\left[\begin{array}{c} c_{ijk}^{\left(1\right)}\\ \vdots \\ c_{ijk}^{\left(n_{T}\right)} \end{array}\right]∼N\left(\left[\begin{array}{c} 0\\ \vdots \\ 0 \end{array}\right],\Sigma_{c}\right),$ in order to take into account part of the correlation between traits not explained by the other random effects. It is interesting to point out that marginally the proposed model is equivalent to a Poisson-lognormal model with random effects. Conditioning on $b_{j}^{\left(l\right)},$ $b_{ij}^{\left(l\right)},$ and $c_{ijk}^{\left(l\right)},$ the distribution of $Y_{ijk}^{\left(l\right)}$ can be approximated using the NB distribution as:

$$\text{P}\left(Y_{ijk}^{\left(l\right)}=y_{ijk}^{\left(l\right)}\right)=\frac{\Gamma \left(y_{ijk}^{\left(l\right)}+r\right)}{y_{ijk}^{\left(l\right)}!\Gamma \left(r\right)}{\left(1-\frac{\mu_{ijk}^{\left(l\right)}}{r+\mu_{ijk}^{\left(l\right)}}\right)}^{r}{\left(\frac{\mu_{ijk}^{\left(l\right)}}{r+\mu_{ijk}^{\left(l\right)}}\right)}^{y_{ijk}^{\left(l\right)}}$$ (2)

with a large enough value of r (for example, r=1000). This probability can be expressed as:

$$\begin{array}{c} \text{P}\left(Y_{ijk}^{\left(l\right)}=y_{ijk}^{\left(l\right)}\right)=\frac{\Gamma \left(y_{ijk}^{\left(l\right)}+r\right)}{y_{ijk}^{\left(l\right)}!\text{Γ}\left(r\right)}{\left(1-\frac{\mu_{ijk}^{\left(l\right)}}{r+\mu_{ijk}^{\left(l\right)}}\right)}^{r}{\left(\frac{\mu_{ijk}^{\left(l\right)}}{r+\mu_{ijk}^{\left(l\right)}}\right)}^{y_{ijk}^{\left(l\right)}}\\ =\frac{\Gamma \left(y_{ijk}^{\left(l\right)}+r\right)}{y_{ijk}^{\left(l\right)}!\Gamma \left(r\right)}\frac{{\left[\exp\left(\eta_{ijk}^{*\left(l\right)}\right)\right]}^{y_{ijk}^{\left(l\right)}}}{{\left[1+\exp\left(\eta_{ijk}^{*\left(l\right)}\right)\right]}^{y_{ijk}^{\left(l\right)}+r}} \end{array}$$

where $\eta_{ijk}^{*\left(l\right)}=x_{i}^{{\left(l\right)}^{T}}\beta^{*\left(l\right)}+b_{j}^{\left(l\right)}+b_{ij}^{\left(l\right)}+c_{ijk}^{\left(l\right)},$ and $\beta^{*\left(l\right)}=\beta^{\left(l\right)}-\log\left(r\right).$ Then, using the identity of Polson *et al.* (2013), this expression (Equation 2) can be expressed as:

$$\begin{array}{l} \text{P}\left(Y_{ijk}^{\left(l\right)}=y_{ijk}^{\left(l\right)}\right)=\frac{\Gamma \left(y_{ijk}^{\left(l\right)}+r\right)}{y_{ijk}^{\left(l\right)}!\Gamma \left(r\right)}\frac{{\left[\exp\left(\eta_{ijk}^{*\left(l\right)}\right)\right]}^{y_{ijk}^{\left(l\right)}}}{{\left[1+\exp\left(\eta_{ijk}^{*\left(l\right)}\right)\right]}^{y_{ijk}^{\left(l\right)}+r}}=\\ \frac{\Gamma \left(y_{ijk}^{\left(l\right)}+r\right)}{y_{ijk}^{\left(l\right)}!\Gamma \left(r\right)}2^{-y_{ijk}^{\left(l\right)}-r}\exp\left(\frac{y_{ijk}^{\left(l\right)}-r}{2}\eta_{ijk}^{*\left(l\right)}\right)\times \overset{\infty }{\underset{0}{\int }}\begin{array}{c} \exp\left(\frac{-\omega_{ijk}^{\left(l\right)}{\left(\eta_{ijk}^{*\left(l\right)}\right)}^{2}}{2}\right)f\left(\omega_{ijk}^{\left(l\right)};y_{ijk}^{\left(l\right)}+r,0\right)d\omega_{ijk}^{\left(l\right)} \end{array} \end{array}$$

where f(⋅ ;b,d) denotes the probability density function of a Pólya-Gamma random variable $\left(\omega_{ijk}^{\left(l\right)}\right)$ with parameters b and d. Then conditioning on $b_{j}^{\left(l\right)},$ $b_{ij}^{\left(l\right)},$ $c_{ijk}^{\left(l\right)},$ and $\omega_{ijk}^{\left(l\right)},$ the distribution of $Y_{ijk}^{\left(l\right)}$ is expressed as:

$$\text{P}\left(Y_{ijk}^{\left(l\right)}=y_{ijk}^{\left(l\right)}|ELSE\right)=\frac{\Gamma \left(y_{ijk}^{\left(l\right)}+r\right)}{y_{ijk}^{\left(l\right)}!\Gamma \left(r\right)}2^{-y_{ijk}^{\left(l\right)}-r}\exp\left(\frac{y_{ijk}^{\left(l\right)}-r}{2}\eta_{ijk}^{*\left(l\right)}\right)\;\exp\left(\frac{-\omega_{ijk}^{\left(l\right)}{\left(\eta_{ijk}^{*\left(l\right)}\right)}^{2}}{2}\right)$$

*ELSE* means that the conditioning was done on $b_{j}^{\left(l\right)},$ $b_{ij}^{\left(l\right)},$ $c_{ijk}^{\left(l\right)},$ and $\omega_{ijk}^{\left(l\right)}.$Therefore, the joint conditional distribution of all phenotypic count responses in the same individual is given by:

$$\text{P}\left(Y_{ijk}^{\left(1\right)}=y_{ijk}^{\left(1\right)},\cdots ,Y_{ijk}^{\left(n_{T}\right)}=y_{ijk}^{\left(n_{T}\right)}|ELSE\right)=\prod_{l=1}^{n_{T}}\text{P}\left(Y_{ijk}^{\left(l\right)}=y_{ijk}^{\left(l\right)}|ELSE\right)=\prod_{l=1}^{n_{T}}\frac{\Gamma \left(y_{ijk}^{\left(l\right)}+r\right)}{y_{ijk}^{\left(l\right)}!\Gamma \left(r\right)}2^{-y_{ijk}^{\left(l\right)}-r}\exp\left(\frac{y_{ijk}^{\left(l\right)}-r}{2}\eta_{ijk}^{*\left(l\right)}-\frac{\omega_{ijk}^{\left(l\right)}{\left(\eta_{ijk}^{*\left(l\right)}\right)}^{2}}{2}\right)$$

$$\propto \exp\left(\sum_{l=1}^{n_{T}}\frac{y_{ijk}^{\left(l\right)}-r}{2}\eta_{ijk}^{*\left(l\right)}-\sum_{t=1}^{n_{T}}\frac{\omega_{ijk}^{\left(l\right)}{\left(\eta_{ijk}^{*\left(l\right)}\right)}^{2}}{2}\right)\;\propto \exp\left(y_{ijk}^{*T}\eta_{ijk}^{*}-\frac{1}{2}\eta_{ijk}^{*T}D_{ijk}\eta_{ijk}^{*}\right)$$ (3)

where $y_{ijk}^{*}=\frac{1}{2}{\left[y_{ijk}^{\left(1\right)}-r,\cdots ,y_{ijk}^{\left(n_{T}\right)}-r\right]}^{T},$ $\eta_{ijk}^{*}={\left[\eta_{ijk}^{\left(1\right)},\cdots ,\eta_{ijk}^{\left(T\right)}\right]}^{T}$ and $D_{ijk}=\mathrm{Diag}\left(\omega_{ijk}^{\left(1\right)},\cdots ,\omega_{ijk}^{\left(n_{T}\right)}\right).$ Here *ELSE* has a similar meaning as before, but adds the corresponding random effects of each response. In this case, we are conditioning on $b_{1j}={\left[b_{j}^{\left(1\right)},\ldots ,b_{j}^{\left(n_{T}\right)}\right]}^{T},$ $b_{2ij}={\left[b_{ij}^{\left(1\right)},\ldots ,b_{ij}^{\left(n_{T}\right)}\right]}^{T},$ $c_{ijk}={\left[c_{ijk}^{\left(1\right)},\ldots ,c_{ijk}^{\left(n_{T}\right)}\right]}^{T},$ and $\omega_{ijk}={\left[\omega_{ijk}^{\left(1\right)},\ldots ,\omega_{ijk}^{\left(n_{T}\right)}\right]}^{T}.$ Therefore, according to Appendix A, the joint conditional distribution for all phenotypic count responses is expressed as:

$$P\left(Y|\beta^{*},b_{1},b_{2},c,\omega \right)\propto \exp\left(y^{*T}\left(X\beta^{*}+Z_{1}b_{1}+Z_{2}b_{2}+c\right)-\frac{1}{2}\;{\left(X\beta^{*}+Z_{1}b_{1}+Z_{2}b_{2}+c\right)}^{T}\times D\left(X\beta^{*}+Z_{1}b_{1}+Z_{2}b_{2}+c\right)\right).$$

where $\omega ={\left[\omega_{1},\ldots ,\omega_{I}\right]}^{T},\omega_{i}={\left[\omega_{i1},\ldots ,\omega_{iJ}\right]}^{T},\omega_{ij}={\left[\omega_{ij1},\ldots ,\omega_{ijK}\right]}^{T}$, and the others terms are defined in the Appendix A. Hereafter we assume that the joint distribution of the random line-trait effects is $b_{1}|\Sigma_{t}∼N_{JL}\left(0,G_{1}\right),$ with $G_{1}=G⊗\Sigma_{t},$ the joint distribution of the interaction random effect of environment i and line j in trait l is$\;b_{2}|\Sigma_{t},\Sigma_{E}∼N_{IJL}\left(0,G_{2}\right),$ with $G_{2}=\Sigma_{E}⊗G⊗\Sigma_{t},\;$and $c|\Sigma_{c}∼N_{JL}\left(0,\Sigma_{c\left(a\right)}\right)$ with $\Sigma_{c(a)}=I_{KJI}⊗\Sigma_{c},$ assuming that all the extra random effects ($c_{ijk}$) are independent and identically distributed. It is important to point out that the correlation between traits is taken into account in both parts of the model: (a) in random effect $b_{1}$ with general covariance matrix $\Sigma_{t},$ and (b) in random effect **c** with general covariance matrix **$\Sigma_{c},$** while the correlation between environments is taken into account in random effect **$b_{2}$** with general covariance matrix $\Sigma_{E}.$ Normal random effect c resulted because we paired a lognormal distribution, exp(c), with the Poisson distribution to create an overdispersed distribution, which is referred to as the Poisson-lognormal distribution. The above statement is clear if we remember that if W has a normal distribution, then Z=exp(W) has a lognormal distribution.

### Prior specification and joint posterior

In this section, we provide the joint posterior density and prior specification for the Bayesian Poisson Multiple-Trait and Multiple-Environment (BPMTME) model. The joint posterior density of the parameter vector becomes:

$$\begin{array}{l} f\left(\beta^{*},b_{1},b_{2},c,\omega ,\Sigma_{t},\Sigma_{E},\Sigma_{c},a,a_{E},a_{c}|Y\right)\\ \propto P\left(Y|\beta^{*},b_{1},b_{2},c,\omega \right)f\left(\beta^{*}\right)f\left(b_{1}|\Sigma_{t}\right)f\left(\Sigma_{t}|a_{1,}\ldots ,a_{L}\right)f\left(a_{1,}\ldots ,a_{L}\right)\\ \times f\left(b_{2}|\Sigma_{t},\Sigma_{E}\right)f\left(\Sigma_{E}|a_{E_{1},}\ldots ,a_{E_{I}}\right)f\left(a_{E_{1},}\ldots ,a_{E_{I}}\right)f\left(c|\Sigma_{c}\right)f\left(\Sigma_{c}|a_{c_{1},}\ldots ,a_{c_{L}}\right)\\ \times f\left(a_{c_{1},}\ldots ,a_{c_{L}}\right)f\left(\omega ;Y+r,0\right) \end{array}$$ (4)

where we are implicitly assuming the following structure of the prior density of the hyper-parameters:

$$\begin{array}{c} f\left(\beta^{*},\Sigma_{t},\Sigma_{E},\Sigma_{c},a,a_{E},a_{c}\right)=f\left(\beta^{*}\right)f\left(\Sigma_{t}|a_{1,}\ldots ,a_{L}\right)\prod_{l=1}^{L}f\left(a_{l}\right)f\left(\Sigma_{E}|a_{E_{1},}\ldots ,a_{E_{I}}\right)\\ \times \prod_{i=1}^{I}f\left(a_{E_{i}}\right)\\ \times f\left(\Sigma_{c}|a_{c_{1},}\ldots ,a_{c_{L}}\right)\prod_{l=1}^{L}f\left(a_{c_{l}}\right). \end{array}$$

More specifically, we assume that $\beta^{*}∼N_{p}\left(\beta_{v}^{*},\Sigma_{v}^{-1}\right),$ $\Sigma_{t}|a_{1,}\ldots ,a_{L}∼IW\left(\nu_{t}+L-1,2\nu_{t}\mathrm{diag}\left(\frac{1}{a_{1}},\ldots \frac{1}{a_{L}}\right)\right),$ $a_{l}∼IG\left(\frac{1}{2},1/A_{l}^{2}\right),\;\;l=1,\ldots ,L,$ $\Sigma_{E}|a_{E1,}\ldots ,a_{L}∼IW\left(\nu_{Ei}+I-1,2\nu_{E}\mathrm{diag}\left(\frac{1}{a_{E1}},\ldots \frac{1}{a_{EL}}\right)\right),$ $a_{Ei}∼IG\left(\frac{1}{2},1/A_{Ei}^{2}\right),$ i=1,…,I, $\Sigma_{c}|a_{c1,}\ldots ,a_{cL}∼IW\left(\nu_{cl}+L-1,2\nu_{c}\mathrm{diag}\left(\frac{1}{a_{c1}},\ldots \frac{1}{a_{cL}}\right)\right),$ and $a_{cl}∼IG\left(\frac{1}{2},1/A_{cl}^{2}\right).$. Here Ω∼IW(κ,B) indicates an inverse Wishart random matrix distribution with density function ${\left|\Omega \right|}^{-\frac{\kappa +p+1}{2}}\exp\left[-\frac{1}{2}tr\left(B\Omega^{-1}\right)\right],$ **κ>0, B, Ω;** both are positive definite matrices, and IG(a,b) denotes an inverse gamma distribution with shape parameter a and rate parameter b.

Using the specified priors we ended up with all full conditional distributions given in Appendix B.

### Gibbs sampler

To produce posterior means for all relevant model parameters, below we outline the exact Gibbs sampler procedure that we propose for estimating the parameters of interest. As is the case with Markov Chain Monte Carlo (MCMC) techniques, the ordering of draws is somewhat arbitrary; however, we suggest the following order:

Step 1. Simulate $\beta^{*}$ according to the normal distribution given in Appendix B (B1).
Step 2. Simulate $b_{1}$ according to the normal distribution given in Appendix B (B2).
Step 3. Simulate $b_{2}$ according to the normal distribution given in Appendix B (B3).
Step 4. Simulate c according to the normal distribution given in Appendix B (B4).
Step 5. Simulate $\Sigma_{t}$ according to the IW distribution given in Appendix B (B5).
Step 6. Simulate $a_{lt}$ according to the IG distribution given in Appendix B (B6).
Step 7. Simulate $\Sigma_{E}$ according to the IW distribution given in Appendix B (B7).
Step 8. Simulate $a_{lE}$ according to the IG distribution given in Appendix B (B8).
Step 9. Simulate $\omega_{ijk}^{\left(l\right)}$ according to the Pólya-gamma distribution given in Appendix B (B9).
Step 10. Simulate $\Sigma_{c}$ according to the IW distribution given in Appendix B (B10).
Step 11. Simulate $a_{lc}$ according to the IG distribution given in Appendix B (B11).
Step 12. Return to step 1 or terminate when chain length is adequate for meeting convergence diagnostics.

### Model implementation

The Gibbs sampler described above for the BPMTME model was implemented as an R-software package (R Core Team 2016). We performed a total of 40,000 iterations; 20,000 samples were used for inference because the first 20,000 were used as burn-in to decrease MCMC errors in prediction accuracy. We used a thinning of five, so that 4000 samples were used for inference. The chain convergence diagnostic was done by visual checks using the trace plots and autocorrelation functions of each component of β coefficients and variance components, and in general we observed that they stabilize very quickly for the real and simulated data. We implemented the prior specification given in the previous section where the BPMTME model was defined. The hyper-parameters used were: $\beta_{v}^{*}=0_{IL},$ $\Sigma_{v}=1\times 10^{4}\Sigma_{0},$ **$\Sigma_{0}=I_{IL},$** $\nu_{t}=\nu_{Ei}=\nu_{cl}=2,$ and $A_{l}=A_{Ei}=A_{cl}=1\times 10^{4},$ where $I_{IL}$ is the identity matrix of dimension IL×IL. All these hyper-parameters were chosen to lead weakly informative priors. Also, it is important to point out that for the implementation with real and simulated data sets, we worked with the sum for each line resulting from its corresponding number of replicates. This was done to save time in the implementation of the proposed model.

### Assessing the prediction accuracy of the models

We used cross-validation 1 (CV1), which mimics a situation where lines were evaluated in some environments for all traits but some lines are missing in other environments, as proposed by Montesinos-López *et al.* (2016b). For real and simulated data, prediction ability was assessed using 10 trn-tst (trn = training and tst = testing) random partitions; we used this approach because it provides higher precision in the predictive estimates than the framework that uses different numbers of folds. However, for the simulated data we used five different partitions (percentages) for the training and testing sets, and the corresponding testing sets were assumed as missed values. The percentages for the training set were 90, 80, 70, 60, and 50% and their corresponding complements were used as testing (tst) sets (tst = 10, tst = 20, tst = 30, tst = 40, and tst = 50%). Of the variety of methods for comparing the predictive posterior distribution to the observed data (generally termed “posterior predictive checks”), we used Spearman’s correlation because the phenotype is not normally distributed. Models with values closer to one indicate better predictions. The predicted observations were calculated with S collected Gibbs samplers as: ${\hat{Y}}^{\left(s\right)}=\exp\left(X{\hat{\beta }}^{*\left(s\right)}+Z_{1}{\hat{\beta }}_{1}^{\left(s\right)}+Z_{2}{\hat{\beta }}_{2}^{\left(s\right)}+\hat{\beta }\right),$ where ${\hat{\beta }}^{*\left(s\right)},{\hat{\beta }}_{1}^{\left(s\right)},{\hat{\beta }}_{2}^{\left(s\right)},\;\text{and}\;\hat{\beta }$ are estimates of $\beta^{*},$ $b_{1},$ $b_{2},$ and c in the *sth* collected sample.

### Simulated data sets

The proposed model was also tested with two simulated data sets (S1 and S2). Both data sets were simulated under the proposed model (described in the statistical model section) with three environments, two traits, 200 genotypes, and five replications. The β coefficients used for both data sets were $\beta^{T}$ = [0.20, 0.25, 0.15, 0.20, 0.30, and 0.32]. The first two β coefficients belong to traits 1 and 2 in environment 1, the third and fourth values belong to traits 1 and 2 in environment 2, and the last two traits for environment 3. The variance-covariance matrices used for the first data set (scenario S1) gave rise to a matrix of correlation between traits and environments of 0.8; these matrices were: $\Sigma_{t}=\left[\begin{array}{cc} 0.005 & 0.0031\\ 0.0031 & 0.003 \end{array}\right],$ **$\Sigma_{E}=\left[\begin{array}{ccc} 0.003 & 0.0022 & 0.0024\\ 0.0022 & 0.0022 & 0.0012\\ 0.0024 & 0.0012 & 0.0030 \end{array}\right],$** and $\Sigma_{c}=\left[\begin{array}{cc} 0.0003 & 0.0003\\ 0.0003 & 0.0004 \end{array}\right].$. The variance-covariance matrices used for the second data set (scenario 2) gave rise to correlation matrices with correlations equal to 0.3; the corresponding covariances were: $\Sigma_{t}=\left[\begin{array}{cc} 0.005 & 0.0012\\ 0.0012 & 0.003 \end{array}\right],$ **$\Sigma_{E}=\left[\begin{array}{ccc} 0.003 & 0.0007 & 0.0009\\ 0.0007 & 0.0022 & 0.0007\\ 0.0009 & 0.0007 & 0.0030 \end{array}\right]$**, and $\Sigma_{c}=\left[\begin{array}{cc} 0.0003 & 0.0001\\ 0.0001 & 0.0004 \end{array}\right].$. It is also important to point out that for both simulated data sets, we assumed independence between genotypes, *i.e.*, $G_{g}=I_{200}.$ The implementation of the BPMTME model for these two simulated data sets was the same as that used with the real data sets described in the section on model implementation.

### Data availability

The Gibbs algorithm described in this paper was implemented in C++ using the Armadillo linear algebra Library (Eddelbuettel and Sanderson 2014). It was included in the R package BMTME as an extension of its current features. With the updated version (0.0.4), the user can also fit count data by means of the same pipeline presented by Montesino-López *et al.* (2016b). However, due to the need for sampling from a Pólya-gamma distribution, it was necessary to include a dependency regarding the R package BayesLogit (Polson *et al.* 2013) and for the moment, we are only distributing the package through CIMMYT’s repository http://hdl.handle.net/11529/10866. Users must have BayesLogit properly installed in their computers before installing BMTME v0.0.4, as well as the R version 3.2.4. or higher. The phenotypic (FHB) and genotypic (marker) data used in this study can also be downloaded from that link.

## Results

The results are given in two sections: the first section gives the results of the simulated data and the second section gives the results of the real data set.

### Prediction accuracy in the simulated data

Table 1 shows the results for both scenarios (S1 and S2) described in the simulation study for the BPMTME model and for the Bayesian Poisson multi-environment (BPME) model (univariate version of the BPMTME model). Since for each scenario we studied the prediction accuracy of the models using 10 random partitions for each of the five training-testing percentages, we provided the average of the Spearman correlation of the 10 random partitions for each testing percentage (10, 20, 30, 40, and 50%) for each scenario and each model (BPMTME and BPME models). First, we compare, for each scenario (S1), the prediction accuracies for each testing percentage between the BPMTME model and the BPME model. Then we compare the prediction accuracy for each testing percentage between the two scenarios.

**Table 1:** Prediction accuracy measured with the average of the 10 random partitions using the Spearman correlation (ASC) for each testing (tst) percentage for the BPMTME and BPME models

		BPMTME					BPME				
		tst = 10%	tst = 20%	tst = 30%	tst = 40%	tst = 50%	tst = 10%	tst = 20%	tst = 30%	tst = 40%	tst = 50%
	Trait–Env	ASC	ASC	ASC	ASC	ASC	ASC	ASC	ASC	ASC	ASC
	11	0.625	0.575	0.710	0.635	0.597	0.480	0.574	0.574	0.551	0.553
	21	0.693	0.568	0.705	0.566	0.542	0.533	0.527	0.560	0.572	0.546
	12	0.815	0.571	0.694	0.615	0.551	0.492	0.395	0.452	0.423	0.397
	22	0.548	0.553	0.685	0.612	0.541	0.597	0.627	0.608	0.609	0.590
	13	0.754	0.575	0.715	0.623	0.518	0.613	0.635	0.616	0.581	0.569
	23	0.610	0.590	0.706	0.628	0.559	0.539	0.601	0.556	0.600	0.597
	Average	0.674	0.572	0.702	0.613	0.551	0.542	0.560	0.561	0.556	0.542
S1		SD	SD	SD	SD	SD	SD	SD	SD	SD	SD
	11	0.118	0.104	0.060	0.045	0.050	0.053	0.018	0.018	0.013	0.016
	21	0.104	0.118	0.046	0.059	0.045	0.033	0.026	0.025	0.013	0.007
	12	0.048	0.113	0.032	0.061	0.051	0.026	0.025	0.016	0.014	0.009
	22	0.136	0.116	0.072	0.065	0.045	0.032	0.032	0.015	0.010	0.011
	13	0.077	0.135	0.045	0.047	0.048	0.033	0.030	0.018	0.012	0.011
	23	0.102	0.118	0.062	0.050	0.043	0.033	0.019	0.014	0.013	0.008
	Trait–Env	Mean	Mean	Mean	Mean	Mean	Mean	Mean	Mean	Mean	Mean
	11	0.728	0.514	0.740	0.643	0.581	0.499	0.581	0.584	0.548	0.561
	21	0.561	0.630	0.711	0.587	0.544	0.511	0.550	0.561	0.584	0.561
	12	0.537	0.623	0.668	0.647	0.567	0.529	0.402	0.437	0.439	0.416
	22	0.531	0.575	0.721	0.625	0.550	0.613	0.641	0.591	0.616	0.600
	13	0.571	0.633	0.674	0.613	0.522	0.607	0.618	0.593	0.564	0.570
	23	0.640	0.538	0.711	0.636	0.568	0.570	0.574	0.544	0.588	0.585
	Average	0.595	0.585	0.704	0.625	0.555	0.555	0.561	0.552	0.557	0.549
S2		SD	SD	SD	SD	SD	SD	SD	SD	SD	SD
	11	0.074	0.122	0.044	0.050	0.054	0.046	0.023	0.016	0.015	0.012
	21	0.154	0.098	0.057	0.055	0.061	0.039	0.028	0.026	0.014	0.007
	12	0.144	0.088	0.041	0.056	0.039	0.023	0.025	0.014	0.013	0.010
	22	0.142	0.108	0.055	0.054	0.035	0.025	0.029	0.020	0.012	0.012
	13	0.137	0.112	0.065	0.058	0.040	0.030	0.027	0.017	0.013	0.010
	23	0.105	0.135	0.064	0.051	0.043	0.032	0.020	0.014	0.014	0.006

SD denotes standard deviations.

The first comparison is important since we hypothesized that the BPMTME model produces better predictions than the BPME model. Under S1, on average, the BPMTME model produced better predictions than the BPME model. For the 10% testing percentage, the BPMTME model was on average 17.89% better than the BPME model. When the testing percentages were 20 and 30%, the BPMTME model was on average 2.1% better than the BPME model. When the testing percentage was 40%, the BPMTME model was on average 9.16% better than the BPME model, and when the testing percentage was 50%, the BPMTME model was on average 1.4% better than the BPME model. It is also important to point out that for trait–environment combination 12, the best model was the BPMTME model for the five testing percentages; the superiority of this model over the BPME model in the five cases was >27%.

For the second scenario (S2) and the 10% testing percentage, the BPMTME model was on average 5.16% better than the BPME model, whereas for the 20, 30, 40, and 50% testing percentages, the BPMTME model was on average 3.17, 21.75, 10.75, and 0.9% better than the BPME model, respectively.

When we compare the prediction accuracies of scenarios S1 and S2, we see that there are no significant differences between the two scenarios, although, on average, for all the testing percentages the BPME model was a little better than BPMTME. To make sense of this result, we need to remember that under scenario S1, the correlation of the three variance-covariance matrices ($\Sigma_{t},\Sigma_{E}$, and **$\Sigma_{c}$**) was 0.8, while under scenario S2 the correlation of these three covariance matrices was 0.30.

### Estimation and prediction accuracy in the real data set

Table 2 gives the parameter estimates of the real data set. The β coefficients between traits and between environments are different, which implies that the counts between environments and between traits are different. The larger counts are observed in environment 3 and the lower counts are observed in environment 1; also, the counts in trait 1 are a little larger in comparison to trait 2. The variances for each trait (that belong to $\Sigma_{t}$) are very similar (0.393 for trait 1 and 0.381 for trait 2) and the correlation between these two traits is 0.948 (with a covariance of 0.367 in $\Sigma_{t}$); that is, the correlation between traits is high since the resulting correlation between traits is close to one. The variances of traits estimated under the variance-covariance matrix ($\Sigma_{c}$) were 0.702 for trait 1 and 0.726 for trait 2, with a correlation of 0.1418 (and a covariance of 0.102); in this case, the correlation between traits that is not explained by $\Sigma_{t}$ is very low (0.1418). However, the variances of environments were very low: 0.0198, 0.003, and 4.94E−05 for environments 1, 2, and 3, respectively (obtained from $\Sigma_{E}$). The correlations between environments were also very low since the correlation between environments 1 and 2 was 0.182 (with covariance 0.0014 from $\Sigma_{E}$), the correlation between environments 1 and 3 was 0.039 (with covariance 3.92E−05 from $\Sigma_{E}$), and the correlation between environments 2 and 3 was 0.037 (with covariance 1.45E−05, from $\Sigma_{E}$). $\Sigma_{c}$ is the general variance-covariance matrix of traits that were not explained by variance-covariance matrix **$\Sigma_{t}.$**

**Table 2:** The posterior means (Mean) and SD of parameter estimates of $\beta^{*},$ $\Sigma_{t},\;\Sigma_{c},\;\text{and}\;\Sigma_{E}$ of the BPMTME model

Coefficient Estimates of $\beta^{*}$			Estimates of $\Sigma_{t}$ and **$\Sigma_{c}$**			Estimates of $\Sigma_{E}$		
Parameter	Mean	SD	Parameter	Mean	SD	Parameter	Mean	SD
${\hat{\beta }}_{11}^{*}$	0.018	0.119	${\hat{\text{Σ}}}_{t11}$	0.393	0.086	${\hat{\text{Σ}}}_{E11}$	0.0198	0.0416
${\hat{\beta }}_{12}^{*}$	−0.151	0.133	${\hat{\text{Σ}}}_{t12}$	0.367	0.063	${\hat{\text{Σ}}}_{E12}$	0.0014	0.0079
${\hat{\beta }}_{21}^{*}$	1.806	0.099	${\hat{\text{Σ}}}_{t22}$	0.381	0.069	${\hat{\text{Σ}}}_{E13}$	3.92E−05	0.0003
${\hat{\beta }}_{22}^{*}$	1.680	0.102	${\hat{\text{Σ}}}_{c11}$	0.702	0.058	${\hat{\text{Σ}}}_{E22}$	0.003	0.0041
${\hat{\beta }}_{31}^{*}$	2.806	0.097	${\hat{\text{Σ}}}_{c12}$	0.102	0.039	${\hat{\text{Σ}}}_{E23}$	1.45E−05	0.0002
${\hat{\beta }}_{32}^{*}$	2.751	0.091	${\hat{\text{Σ}}}_{c22}$	0.726	0.060	${\hat{\text{Σ}}}_{E33}$	4.94E−05	0.0001

SD denotes standard deviations.

Table 3 shows that, in general, there are no significant differences in terms of prediction accuracy between the two models (BPMTME and BPME); however, it is very interesting that predictions are pretty high under both models, since in all cases the predictions are >0.5. For example, under both models the lowest predictions were for trait–environment combination 11, while the best predictions were for trait–environment combinations 13 and 23.

**Table 3:** Prediction accuracy of the real data set measured based on the average of the 10 random partitions using the Spearman correlation (ASC) for each testing (tst) percentage for the BPMTME and BPME models in bold are the best predictions.

	BPMTME		BPME	
Trait–Env	Mean	SE	Mean	SE
11	0.5008	0.0666	**0.5145**	0.0595
21	0.5309	0.0375	**0.5814**	0.0290
12	0.6805	0.0287	**0.7064**	0.0383
22	**0.7025**	0.0462	0.6715	0.0394
13	0.7447	0.0158	**0.7672**	0.0195
23	**0.7576**	0.0262	0.7187	0.0214
Average	0.6528	0.0368	0.6600	0.0345

SE denotes the standard error.

**Figure 1:**
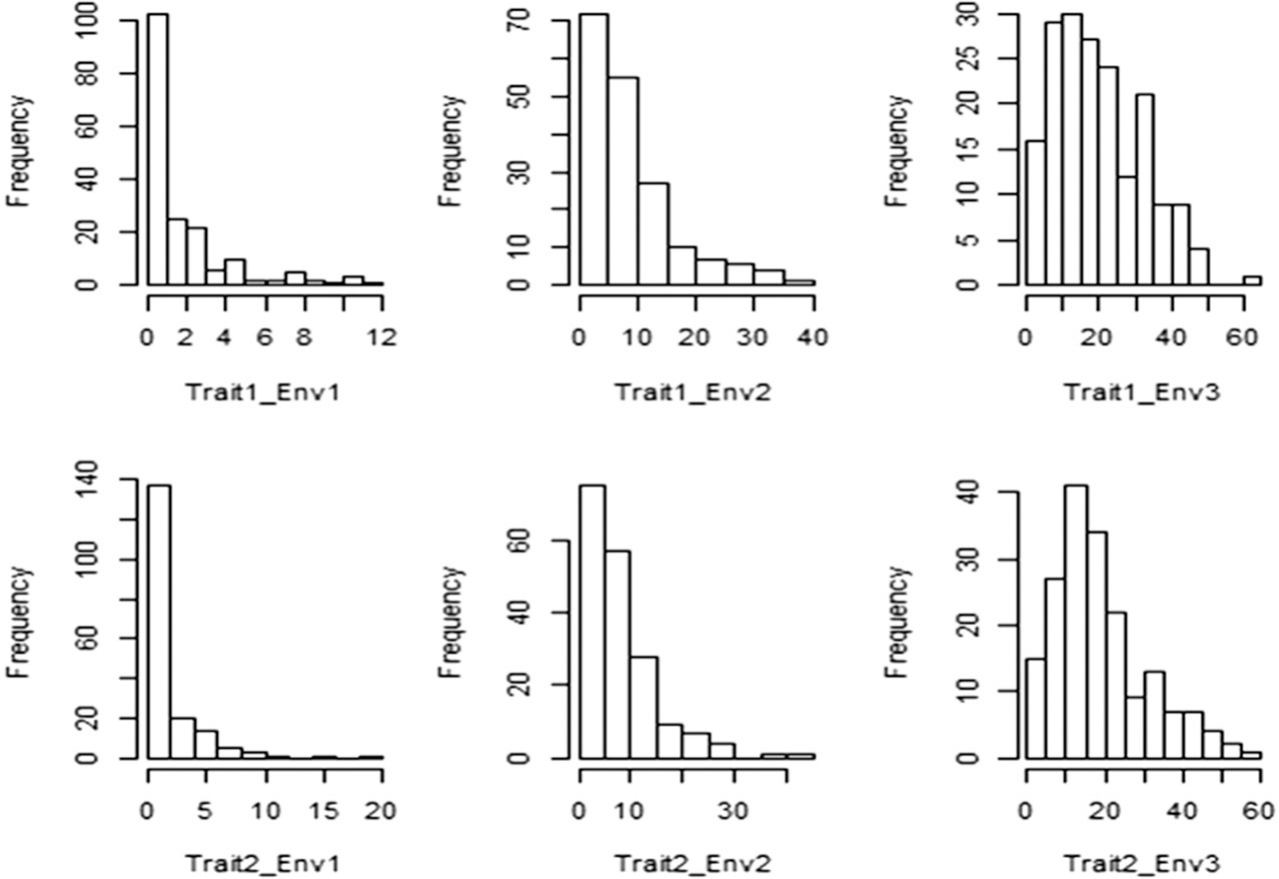
Histogram of count frequencies of the real data set for the two traits under study for each environment.

These results can be explained in part because the correlation observed between traits in correlation matrix $\Sigma_{c}$ was very low (0.1418), and because the correlation observed in the correlation matrix between environments was also low (see Table 2). However, although the correlation between traits observed in correlation matrix $\Sigma_{t}$ was very high (0.948), this alone did not contribute to superior predictions under the BPMTME model in comparison to the BPME model. It is important to point out that these results with the real data do not indicate that the BPMTME model is not useful, but rather that there is no advantage in using this model over the BPME model when the correlation structures of traits ($\Sigma_{t},$ **$\Sigma_{c}$**) and environments ($\Sigma_{E}$) are not high. Another possible explanation of why the BPMTME model did not outperform the BPME model is that the number of wheat lines in this data set is limited (only 182) and only 18 (10%) of them are predicted in some environments. When our proposed BPMTME model is used with limited data sets, it is likely to capture idiosyncrasies of the data rather than the true underlying processes; this problem may be entirely due to limitations the data impose on our ability to detect the underlying processes, rather than to any inherent value of simple models. Although the results in Table 3 do not favor the BPMTME model, we can argue that the proposed multivariate model has a low risk of doing worse in terms of prediction accuracy. For this reason, we are convinced that when dealing with prediction of count phenotypes for multiple traits and multiple environments, the savviest solution is to run both models and choose the one with better prediction performance. Another possible reason for the difference in performance of both models may be the presence of more zeros than our model can support in the phenotypes under study, since Figure 1 shows that for trait–environment combinations, Trait1_Environment1, Trait1_Environment2, Trait2_Environment1, and Trait2_Environment2, there is overdispersion for the excess zeros. In the statistical literature, zero-inflated and hurdle models have been proposed for coping with zero-inflated outcome data with or without overdispersion. For this reason, as one reviewer suggested, the proposed models for multiple-traits and multiple-environments can be expanded to deal with the high occurrence of zeros in the observed dependent variables to end up with zero-inflated and hurdle versions for multiple-traits and multiple-environments.

## Discussion

In this paper, we propose a Poisson-lognormal multivariate model that takes into account the T×E, T×G, and T×G×E interaction terms, and helps to improve prediction capacity in comparison to the univariate Poisson-lognormal model, which only takes into account the G×E interaction term. The proposed model is original in the sense that an exact Gibbs sampler was obtained, which makes this model more efficient than existing Poisson-lognormal multivariate models that do not take into account interaction terms and use the Metropolis-Hastings algorithm to estimate parameters. Also, the lognormal random effect exp(c) that was introduced in the linear predictor allows having an approximate Poisson-lognormal model. The advantage of introducing these lognormal random effects was twofold: (1) accounting for overdispersion (more than the Poisson variance) and (2) exploiting the correlation between traits that was not captured by the variance-covariance matrix of traits ($\Sigma_{t}$). We introduced a general covariance structure between the traits for the extra random effect $\left(\Sigma_{c}\right),$ which plays a really important role because it also allows borrowing information between traits. Additionally, the proposed model also has a general covariance structure between environments which also allows borrowing information between environments. It is important to point out that if a gamma distribution was assumed for the random effect c, this would produce a multivariate negative binomial model which, in addition to being very inefficient for estimating the scale parameter, only allows positive correlation structures because the NB model (univariate and multivariate) was motivated solely for mathematical convenience.

Another advantage of the proposed BPMTME model is that it can be used for performing univariate analysis employing the predictor given in Equation (1), using the appropriate design matrices of environments, lines, and genotype by environment. This alternative is really helpful since in addition to implementing the multiple-trait and multi-environment model for count data, it also allows implementing a univariate multiple-environment model with the same proposed code under a Poisson-lognormal model. Here it is important to point out that the resulting univariate Poisson-lognormal model is different from the Poisson and NB models with the G×E interaction term proposed by Montesinos-López *et al.* (2016a), given that the models with G×E proposed by these authors do not have the normal random effect $c_{ijk}^{\left(l\right)}.$ For this reason, a comparison with this model was not included. However, according to the existing statistical literature, there is evidence that when there is overdispersion, the Poisson-lognormal model does a better job than the Poisson only model.

According to our results, the proposed model may be useful for breeders interested in modeling more than one count trait in many environments simultaneously. Although in the simulation study the BPMTME model was better in terms of prediction accuracy than the BPME model, in the real data set both models had similar prediction performance. Although the proposed BPMTME model showed no superiority in the real data set, we believe that when the correlations between traits and between environments are high, the proposed model should be superior. However, more empirical evidence is necessary to be sure that the proposed model helps to increase prediction accuracy under these circumstances.

As previously mentioned, our results with the real data sets indicated that the univariate model was slightly better than our multivariate model in terms of prediction accuracy. This result, which was not expected, has been documented in areas like econometrics where multivariate models have been better at modeling interdependencies and achieve better fit. Within a given sample, it has been found that univariate methods outperform multivariate methods in sample predictions. Some of the reasons that may explain these results are given here. (a) Multivariate models have more parameters than univariate models (parsimonious). Every additional parameter is an unknown quantity and has to be estimated. This estimation brings an additional source of error due to sampling variation. (b) The number of potential candidates for multivariate models exceeds its univariate counterpart. Model selection is therefore more complex, lengthier, and more prone to errors, which affect prediction. (c) It is difficult to generalize nonlinear procedures in the multivariate case. Generally, multivariate models must have a simpler structure than univariate ones to overcome the additional complexity of being multivariate. For example, while a researcher may use a nonlinear model for univariate data, she/he may refrain from using the multivariate counterpart or such a generalization may not have been developed. Then, multivariate models will miss the nonlinearities that are handled properly by the univariate models. (d) Outliers can have a more serious effect on multivariate predictions than on univariate predictions. Moreover, it is easier to spot and control outliers in the univariate context, *etc*. For these reasons, we believe it is necessary to explore other approaches to prediction when dealing with multivariate responses (count and normal phenotypes) and multiple environments, for when the number of traits, lines, and environments increased significantly even with normal phenotypes, the implementation of this model became very difficult.

Finally, although the proposed model has some advantages, it also has some disadvantages. The main disadvantage of the proposed BPMTME model is that it is computationally demanding. Although we implemented this model in C++ code, when the number of lines, environments, and traits grows substantially, the time required for its implementation increases greatly and can be untenable for large numbers. The second disadvantage is that our proposed BPMTME model is in reality an approximation to the true BPMTME model: instead of working directly with the Poisson model, we approximated it with the negative NB distribution using r=1000  as a scale parameter, which works very well for small counts, as was shown by Montesinos-López *et al.* (2016a). Thanks to this approximation, it was possible to obtain an exact Gibbs sampler that is more powerful for complex and large data sets.

### Conclusions

In this paper, we propose a multiple-trait, multiple-environment model for count data under an approximate Bayesian Poisson-lognormal model. The Gibbs sampler obtained is an approximation since the Poisson-lognormal model is approximated using the NB distribution in conjunction with a lognormal random effect. The proposed BPMTME model was compared with its univariate counterpart (BPME), and in the simulation examples, the proposed multivariate model (BPMTME) was considerably better in terms of prediction accuracy. This can be expected when there is a moderate or large correlation between traits, which allows borrowing information between traits. However, the prediction accuracy of the proposed multivariate model was not superior to that of the univariate model with the real data set. The proposed BPMTME model allows borrowing information between environments because it takes into account a general covariance matrix between environments. For these reasons, we believe that more empirical work is needed to determine whether the proposed BPMTME model is really a better tool than the BPME model tool for breeders interested in simultaneously improving more than one count trait in many environments. However, although an exact Gibbs sampler was proposed and the program was implemented in C++, it is computationally demanding and more work is required for increasing its speed. Nonetheless, we are convinced that this limitation will disappear or diminish considerably in the coming years as computer science is improving very quickly.

## Appendix A: Joint Distribution of All Phenotypic Count Responses

Conditioning on $b_{1j},$ $b_{2ij},$ $c_{ij}={\left[c_{ij1},\ldots ,c_{ijK}\right]}^{T}$ and $\omega_{ij}={\left[\omega_{ij1},\ldots ,\omega_{ijK}\right]}^{T},$ the joint conditional distribution for all the replications in environment i and genotype j is

$${\text{L}}_{ij}=\prod_{k=1}^{K}{\text{L}}_{ijk}\propto \prod_{l=1}^{n_{T}}\exp\left(\sum_{k=1}^{K}y_{ijk}^{*T}\eta_{ijk}^{*}-\frac{1}{2}\sum_{k=1}^{K}\eta_{ijk}^{*T}D_{ijk}\eta_{ijk}^{*}\right)=\exp\left(y_{ij}^{*T}\eta_{ij}^{*}-\frac{1}{2}\eta_{ij}^{*T}D_{ij}\eta_{ij}^{*}\right)$$

where $y_{ij}^{*}={\left[y_{ij1}^{*T},\cdots ,y_{ijK}^{*T}\right]}^{T},\;\eta_{ij}^{*}={\left[\eta_{ij1}^{*T},\cdots ,\eta_{ijK}^{*T}\right]}^{T}\text{and}\;D_{ij}=\mathrm{diag}\left(D_{ij1},\cdots ,D_{ijK}\right).$ Then, the joint conditional distribution for all the observations in environment i is given by

$${\text{L}}_{i}=\prod_{j=1}^{J}{\text{L}}_{ij}\propto \exp\left(y_{i}^{*T}\eta_{i}^{*}-\frac{1}{2}\eta_{i}^{*T}D_{i}\eta_{i}^{*}\right)$$

where $y_{i}^{*}={\left[y_{i1}^{*T},\cdots ,y_{iJ}^{*T}\right]}^{T},\;\eta_{i}^{*}={\left[\eta_{i1}^{*T},\cdots ,\eta_{iJ}^{*T}\right]}^{T}\text{, and}\;D_{i}=\mathrm{diag}\left(D_{i1},\cdots ,D_{iJ}\right).$ Finally, the joint conditional distribution for all the observations is

$$\text{L}=\prod_{i=1}^{I}{\text{L}}_{i}\propto \exp\left(y^{*T}\eta^{*}+\frac{1}{2}\;\eta_{i}^{*T}D\;\eta^{*}\right)$$

where $y*={\left[y_{1}^{*T},\cdots ,y_{I}^{*T}\right]}^{T},\;\eta_{i}^{*}={\left[\eta_{1}^{*T},\cdots ,\eta_{I}^{*T}\right]}^{T}\text{, and}\;D=\mathrm{diag}\left(D_{1},\cdots ,D_{I}\right).$

Note that the predictor (1) can be expressed as

$$\eta_{ijk}^{*\left(l\right)}=\beta_{i\;}^{\left(l\right)}+b_{1j}^{\left(l\right)}+b_{2ij}^{\left(l\right)}+c_{ijk}^{\left(l\right)}-\log\left(r\right)=\beta_{i\;}^{*\left(l\right)}+b_{1j}^{\left(l\right)}+b_{2ij}^{\left(l\right)}+c_{ijk}^{\left(l\right)}=\;x_{i}^{\left(l\right)T}\beta_{\;}^{*\left(l\right)}+b_{1j}^{\left(l\right)}+b_{2ij}^{\left(l\right)}+c_{ijk}^{\left(l\right)}$$

with $\beta^{*\left(l\right)T}=\left[\beta_{1}^{*\left(l\right)},\cdots ,\beta_{I}^{*\left(l\right)}\right],\;\;x_{i}^{\left(l\right)T}=\left[x_{i1}^{\left(l\right)},\cdots ,x_{iI}^{\left(l\right)}\right]$ and where $x_{ii^{\prime }}^{\left(l\right)}=1$ if $i=i^{\prime }$ and $x_{ii^{\prime }}^{\left(l\right)}=0$ otherwise. This implies that

$$\eta_{ijk}^{*}=\left[\begin{array}{c} \eta_{ijk}^{*(1)}\\ \vdots \\ \eta_{ijk}^{*(n_{T})} \end{array}\right]=X_{ijk}\beta^{*}+b_{1j}+b_{2ij}+c_{ijk},$$

where $X_{ijk}=\left[\begin{array}{c} e_{1}^{T}⊗x_{i}^{{\left(1\right)}^{T}}\\ \vdots \\ e_{n_{T}}^{T}⊗x_{i}^{{\left(n_{T}\right)}^{T}} \end{array}\right],$ where $e_{l}^{T}$ is the l-th element of the canonical basis of ${\mathbb{R}}^{n_{T}}.$ $\beta^{*}=\left[\begin{array}{c} \beta^{*\left(1\right)}\\ \vdots \\ \beta^{*\left(n_{T}\right)} \end{array}\right],$ $b_{1j}=\left[\begin{array}{c} b_{1j}^{\left(1\right)}\\ \vdots \\ b_{1j}^{\left(n_{T}\right)} \end{array}\right],$ $b_{2ij}=\left[\begin{array}{c} b_{2ij}^{\left(1\right)}\\ \vdots \\ b_{2ij}^{\left(n_{T}\right)} \end{array}\right],$ and $c_{ijk}=\left[\begin{array}{c} c_{ijk}^{\left(1\right)}\\ \vdots \\ c_{ijk}^{\left(n_{T}\right)} \end{array}\right].$. From here,

$$\eta_{ij}^{*}=\left[\begin{array}{c} \eta_{ij1}^{*}\\ \vdots \\ \eta_{ijK}^{*} \end{array}\right]=X_{ij}\beta^{*}+Z_{1ij}b_{1j}+Z_{2ij}b_{2ij}+c_{ij},$$

where $X_{ij}=\left[\begin{array}{c} X_{ij1}\\ \vdots \\ X_{ijK} \end{array}\right],$ $Z_{1ij}=1_{K}⊗I_{n_{T}},$ $Z_{2ij}=1_{K}⊗I_{n_{T}},$ $c_{ij}=\left[\begin{array}{c} c_{ij1}\\ \vdots \\ c_{ijK} \end{array}\right],$ and $1_{K}$ is a column vector of ones of dimension K.

This implies that

$$\eta_{i}^{*}=\left[\begin{array}{c} \eta_{i1}^{*}\\ \vdots \\ \eta_{iJ}^{*} \end{array}\right]=X_{i}\beta^{*}+Z_{1i}b_{1}+Z_{2i}b_{2i}+c_{i}$$

where $X_{i}=\left[\begin{array}{c} X_{i1}\\ \vdots \\ X_{iJ} \end{array}\right],\;Z_{1i}=\left[\begin{array}{ccc} Z_{1i1} & \cdots & 0\\ \vdots & \ddots & \vdots \\ 0 & \cdots & Z_{1iJ} \end{array}\right],$ $Z_{2i}=\left[\begin{array}{ccc} Z_{2i1} & \cdots & 0\\ \vdots & \ddots & \vdots \\ 0 & \cdots & Z_{2iJ} \end{array}\right],$ $b_{1}=\left[\begin{array}{c} b_{11}\\ \vdots \\ b_{1J} \end{array}\right],$ $b_{2i}=\left[\begin{array}{c} b_{2i1}\\ \vdots \\ b_{2iJ} \end{array}\right],$ and $c_{i}=\left[\begin{array}{c} c_{ij}\;\\ \vdots \\ c_{iJ} \end{array}\right].$. Then

$$\eta^{*}=\left[\begin{array}{c} \eta_{1}^{*}\\ \vdots \\ \eta_{I}^{*} \end{array}\right]=X\beta^{*}+Z_{1}b_{1}+Z_{2}b_{2}+c$$

where $X=\left[\begin{array}{c} X_{1}\\ \vdots \\ X_{I} \end{array}\right],$ $Z_{1}=\left[\begin{array}{c} Z_{11}\\ \vdots \\ Z_{1I} \end{array}\right],$ $Z_{2}=\left[\begin{array}{ccc} Z_{21} & \cdots & 0\\ \vdots & \ddots & \vdots \\ 0 & \cdots & Z_{2I} \end{array}\right],$ $b_{2}=\left[\begin{array}{c} b_{21}\\ \vdots \\ b_{2I} \end{array}\right],$ and $c=\left[\begin{array}{c} c_{1}\;\\ \vdots \\ c_{I} \end{array}\right].$. Finally,

$$L=\exp\left(y^{*T}\eta^{*}-\eta^{*T}D\eta^{*}\right)\propto \exp\left(y^{*T}\left(X\beta^{*}+Z_{1}b_{1}+Z_{2}b_{2}+c\right)-\frac{1}{2}\;{\left(X\beta^{*}+Z_{1}b_{1}+Z_{2}b_{2}+c\right)}^{T}D\left(X\beta^{*}+Z_{1}b_{1}+Z_{2}b_{2}+c\right)\right).$$

### Full conditional for $\beta^{*}$

$$f\left(\beta^{*}|-\right)\propto \;\text{exp}\left(y^{*T}\left(X\beta^{*}\right)-\frac{1}{2}\;{\left(X\beta^{*}+Z_{1}b_{1}+Z_{2}b_{2}+c\right)}^{T}D\left(X\beta^{*}+Z_{1}b_{1}+Z_{2}b_{2}+c\right)-\frac{1}{2}{\left(\beta^{*}-\beta_{v}^{*}\right)}^{T}\Sigma_{v}^{-1}\left(\beta^{*}-\beta_{v}^{*}\right)\right)$$

$$\begin{array}{c} \propto \exp\left(-\frac{1}{2}\beta^{*T}\left(\Sigma_{v}^{-1}+X^{T}\mathrm{DX}\right)\beta^{*}-\frac{1}{2}\;\left(-2\right)\left[\beta_{v}^{*T}\Sigma_{v}^{-1}+y^{*T}X-{\left(Z_{1}b_{1}+Z_{2}b_{2}+c\right)}^{T}\mathrm{DX}\right]\beta^{*}\right)\\ \propto \;\text{exp}\left(-\frac{1}{2}\beta^{*T}{\left(\Sigma_{v}^{*}\right)}^{-1}\beta^{*}-\frac{1}{2}\;\left(-2\right)\beta_{v}^{**T}{\left(\Sigma_{v}^{*}\right)}^{-1}\beta^{*}\right)\; \end{array}$$

$$\propto \exp\left[\frac{1}{2}{\left(\beta^{*}-\beta_{v}^{**}\right)}^{T}{\left(\Sigma_{v}^{*}\right)}^{-1}\left(\beta^{*}-\beta_{v}^{**}\right)\right]∼\text{N}\left(\beta_{v}^{**},\Sigma_{v}^{*}\right)$$ (B1)

where $\Sigma_{v}^{*}={\left(\Sigma_{v}^{-1}+X^{T}\mathrm{DX}\right)}^{-1}$ and $\beta_{v}^{**}=\Sigma_{v}^{*}\left[\Sigma_{v}^{-1}\beta_{v}^{*}+X^{T}y^{*}-X^{T}D\left(Z_{1}b_{1}+Z_{2}b_{2}+c\right)\right].$

### Full conditional for$\;b_{1}$

$$\begin{array}{c} f\left(b_{1}|-\right)\propto \text{exp}\left(y^{*T}Z_{1}b_{1}-\frac{1}{2}\eta^{*T}{\mathrm{D\eta}}^{*}-\frac{1}{2}b_{1}^{T}G_{1}^{-1}b_{1}\right)\;\\ \propto \exp\left(-\frac{1}{2}b_{1}^{T}\left(G_{1}^{-1}+Z_{1}^{T}DZ_{1}\right)b_{1}-\frac{1}{2}\;\left(-2\right)\left[y^{*T}Z_{1}-{\left(\mathrm{X\beta}*+Z_{2}b_{2}+c\right)}^{T}DZ_{1}\right]b_{1}\right)\;\;\\ \propto \exp\left(-\frac{1}{2}b_{1}^{T}\left(G_{1}^{*}\right)b_{1}-\frac{1}{2}\;\left(-2\right)b_{1}{\left(G_{1}^{*}\right)}^{-1}b_{1}\right)\; \end{array}$$

$$\propto \exp\left(-\frac{1}{2}{\left(b_{1}-b_{1}^{*}\right)}^{T}{\left(G_{1}^{*}\right)}^{-1}\left(b_{1}-b_{1}^{*}\right)\right)∼N\left(b_{1}^{*},G_{1}^{*}\right)$$ (B2)

where **$G_{1}^{*}={\left(G_{1}^{-1}+Z_{1}^{T}\mathrm{DZ}\right)}^{-1}$** and $b_{1}^{*}=G_{1}^{*}{\left(Z_{1}^{T}y^{*}-Z_{1}^{T}D\left(X\beta^{*}+Z_{2}b_{2}+c\right)\right)}^{}.$

### Full conditional for$\;b_{2}$

In a similar way as the full conditional of $\;b_{1},\;$ then

$$b_{2}|∼N\left(b_{2}^{*},G_{2}^{*}\right)$$ (B3)

where $G_{2}^{*}={\left(G_{2}^{-1}+Z_{2}^{T}DZ_{2}\right)}^{-1}$ and $b_{2}^{*}=G_{2}^{*}\left(Z_{2}^{T}y^{*}-Z_{2}^{T}D\left(X\beta^{*}+Z_{1}b_{1}+c\right)\right).$

### Full conditional for c

First note that c is equal to $c={\left[\begin{array}{ccc} c_{1} & \ldots & c_{I} \end{array}\right]}^{T}={\left[\begin{array}{ccc} c_{11}\ldots c_{1J}\ldots c_{I1} & \ldots & c_{IJ} \end{array}\right]}^{T},$ and we need to remember that $c_{ijk}={\left[\begin{array}{ccc} c_{ijk}^{\left(1\right)} & \ldots & c_{ijk}^{\left(n_{T}\right)} \end{array}\right]}^{T}∼N\left(0,\Sigma_{c}\right),$ for all i, j, and k. This implies that $c_{ij}={\left[\begin{array}{ccc} c_{ij1} & \ldots & c_{ijK} \end{array}\right]}^{T}\;\;∼N\left(0,I_{\text{K}}⊗\Sigma_{c}\right),$ $c_{i}={\left[\begin{array}{ccc} c_{i1} & \ldots & c_{iJ} \end{array}\right]}^{T}\;∼N\left(0,I_{KJ}⊗\Sigma_{c}\right),$ and $c={\left[\begin{array}{ccc} c_{1} & \ldots & c_{I} \end{array}\right]}^{T}\;∼N\left(0,\Sigma_{c(a)}\right),$ with $\Sigma_{c(a)}=I_{KJI}⊗\Sigma_{c}.$ Therefore,

$$\begin{array}{c} f\left(c|-\right)\propto \text{exp}\left(y^{*T}c-\frac{1}{2}\eta^{*T}{\mathrm{D\eta}}^{*}-\frac{1}{2}c^{T}\Sigma_{c\left(a\right)}^{-1}c\right)\;\\ \propto \exp\left(-\frac{1}{2}c^{T}\left(\Sigma_{c\left(a\right)}^{-1}+D\right)c-\frac{1}{2}\left(-2\right)\left[y^{*T}-{\left(\mathrm{X\beta}*+Z_{1}b_{1}+Z_{2}b_{2}\right)}^{T}D\right]c\right)\;\\ \propto \exp\left(-\frac{1}{2}{\left(c-c*\right)}^{T}{\left(\Sigma_{c\left(a\right)}^{*}\right)}^{-1}\left(c-c*\right)\right)∼N\left(c*,\Sigma_{c\left(a\right)}^{*}\right)\;\; \end{array}$$ (B4)

where $\Sigma_{c(a)}^{*}={\left(\Sigma_{c(a)}^{-1}+D\right)}^{-1}$ and $c^{*}=\Sigma_{c(a)}^{*}\left[y*-D\left(X\beta^{*}+Z_{1}b_{1}+Z_{2}b_{2}\right)\right].$

### Full conditional for $\Sigma_{t}$

$$f\left(\Sigma_{t}|-\right)\propto f\left(b_{1}|\Sigma_{t}\right)f\left(b_{2}|\Sigma_{E},\Sigma_{t}\right)f\left(\Sigma_{t}|a_{t}\right)\propto \;\frac{1}{{\left|\Sigma_{t}\right|}^{J/2}}\frac{1}{{\left|\Sigma_{t}\right|}^{IJ/2}}\exp\left(-\frac{1}{2}b_{1}^{T}G_{1}^{-1}b_{1}-\frac{1}{2}b_{2}^{T}G_{2}^{-1}b_{2}\right)f\left(\Sigma_{t}|a_{t}\right)\;$$

$$\propto \;\frac{1}{{\left|\Sigma_{t}\right|}^{\frac{J+IJ}{2}}}\text{exp}\left(-\frac{1}{2}tr\left(\Sigma_{t}^{-1}\left(B_{1}^{*}G^{-1}B_{1}^{*T}+B_{2}^{*}G_{3}^{-1}B_{2}^{*T}\right)\right)\right){\left|C_{t}\right|}^{\frac{v_{t}+n_{t}-1}{2}}{\left|\Sigma_{t}\right|}^{\frac{v_{t}+n_{t}}{2}}\exp\left(-\frac{1}{2}tr\left(C_{t}\Sigma_{t}^{-1}\right)\right)\propto \;{\left|\Sigma_{t}\right|}^{-\frac{v_{t}+J\left(1+I\right)+2n_{t}}{2}}\exp\left(-\frac{1}{2}tr\left(C_{t}^{*}\Sigma_{t}^{-1}\right)\right)$$

$$∼IW\left(v=v_{t}+\left(1+I\right)J+n_{T}-1,C_{t}^{*}\right)$$ (B5)

where $C_{t}^{*}=C_{t}+B_{1}^{*}G_{g}^{-1}B_{1}^{*T}+B_{2}^{*}G_{3}^{-1}B_{2}^{*T}$ and $C_{t}=2v_{t}\mathrm{diag}\left(\begin{array}{ccc} \frac{1}{a_{1t}} & \cdots & \frac{1}{a_{n_{T}t}} \end{array}\right),$ and $B_{1}^{*},$ $B_{2}^{*},$ and $G_{3}^{-1}$ are matrices defined below. To see this, note that $b_{1}^{T}G_{1}^{-1}b_{1}={\left\|G_{1}^{-1/2}b_{1}\right\|}^{2}$ and $G_{1}^{-1/2}=G^{-1/2}⊗\Sigma_{t}^{-\left(1/2\right)}.$ Using the property $\left(B^{T}⊗A\right)\mathrm{vec}\left(X\right)=\mathrm{vec}\left(\mathrm{AXB}\right),$ we have that $G_{1}^{-1/2}b_{1}=\left(G^{-1/2}⊗\Sigma_{t}^{-\left(1/2\right)}\right)b_{1}=\left(G^{-1/2}⊗\Sigma_{t}^{-\left(1/2\right)}\right)\mathrm{vec}\left(B_{1}^{*}\right)=\mathrm{vec}\left(\Sigma_{t}^{-1/2}B_{1}^{*}G^{-1/2}\right)=\left[\begin{array}{c} \Sigma_{t}^{-\left(1/2\right)}d_{1}\\ \vdots \\ \Sigma_{t}^{-1/2}d_{J} \end{array}\right],$ where $B_{1}^{*}=\left[b_{11}\cdots b_{1J}\right]$ and $d_{j},\;j=1,\;\ldots ,\;J,$ are the columns of $B_{1}^{*}G^{-1/2}.$ Then $b_{1}^{T}G_{1}^{-1}b_{1}=\sum_{j=1}^{J}d_{j}^{T}\Sigma_{t}^{-1/2}\Sigma_{t}^{-1/2}d_{j}=tr\left(\Sigma_{t}^{-1}\sum_{j=1}^{J}d_{j}d_{j}^{T}\right)=tr\left(\Sigma_{t}^{-1}B_{1}^{*}G^{-1/2}G^{-1/2}B_{1}^{*T}\right)=tr\left(B_{1}^{*}G^{-1}B_{1}^{*T}\Sigma_{t}^{-1}\right).$ Defining $G_{3}=\Sigma_{E}⊗G,$ in a similar way, we see that

$$b_{2}^{T}G_{2}^{-1}b_{2}=tr\left(\Sigma_{t}^{-1}B_{2}^{*}G_{3}^{-1}B_{2}^{*T}\right)=tr\left(B_{2}^{*}G_{3}^{-1}B_{2}^{*T}\Sigma_{t}^{-1}\right),$$

where $B_{2}^{*}=\left[b_{211},\cdots ,b_{21J},\cdots ,b_{2I1},\cdots ,b_{2IJ}\right].$

### Full conditional for $a_{lt}$

$$\begin{array}{c} f\left(a_{lt}|ELSE\right)\propto f\left(\Sigma_{t}|a_{lt}\right)f\left(a_{lt}\right)\propto \frac{1}{{\left(a_{l}\right)}^{\frac{\nu_{t}+n_{T}}{2}+1}}\exp\left(-\frac{\frac{1}{A_{lt}^{2}}+\nu_{t}{\left(\Sigma_{t}^{-1}\right)}_{ll}}{a_{lt}}\right)\\ \propto IG\left(\frac{\nu_{t}+n_{T}}{2},1/A_{lt}^{2}+\nu_{t}{\left(\Sigma_{t}^{-1}\right)}_{ll}\right) \end{array}$$ (B6)

### Full conditional for $\Sigma_{E}$

$$f\left(\Sigma_{E}|-\right)\propto \;\frac{1}{{\left|\Sigma_{E}\right|}^{\frac{n_{T}J}{2}}}\exp\left(-\frac{1}{2}tr\left(B_{2}^{*T}G_{1}^{-1}B_{3}^{*}\Sigma_{E}^{-1}\right)\right)\frac{1}{{\left|\Sigma_{E}\right|}^{\frac{v_{E}+2I}{2}}}\exp\left[-\frac{1}{2}tr\left(C_{E}\Sigma_{E}^{-1}\;\right)\right]\propto \;\frac{1}{{\left|\Sigma_{E}\right|}^{\frac{v_{E}+n_{T}J+2I}{2}}}\text{exp}\left[-\frac{1}{2}tr\left(C_{E}^{*}\Sigma_{E}^{-1}\right)\right]\;\propto \;{\left|\Sigma_{E}\right|}^{-\frac{v_{E}+n_{T}J+I+I-1+1}{2}}\exp\left[-\frac{1}{2}tr\left(C_{E}^{*}\Sigma_{E}^{-1}\right)\right]$$

$$∼IW\left(v_{E}+n_{T}J+I-1,C_{E}^{*}\right)$$ (B7)

where $C_{E}^{*}=C_{E}+B_{3}^{*T}G_{1}^{-1}B_{3}^{*}$ and $B_{3}^{*}$ is defined below. First, this is because $b_{2}^{T}\left(\Sigma_{E}^{-1}⊗G_{1}^{-1}\right)b_{2}={\left\|\left(\Sigma_{E}^{-1/2}⊗G_{1}^{-1/2}\right)b_{2}\right\|}^{2}$ and $\left(\Sigma_{E}^{-1/2}⊗G_{1}^{-1/2}\right)b_{2}=\left(\Sigma_{E}^{-1/2}⊗G_{1}^{-1/2}\right)\mathrm{vec}\left(B_{3}^{*}\right)=\mathrm{vec}\left(G_{1}^{-1/2}B_{2}^{*}\Sigma_{E}^{-1/2}\right),$ where $B_{3}^{*}$ is a matrix with I columns such that $\mathrm{vec}\left(B_{3}^{*}\right)=b_{2}.$ Then, expressing$G_{1}^{-1/2}B_{3}^{*}\Sigma_{E}^{-1/2}=\left[f_{1},\cdots ,f_{I}\right],$ $b_{2}^{T}G_{1}^{-1}b_{2}=\sum_{i=1}^{I}f_{i}^{T}f_{i}=\sum_{i=1}^{I}e_{i}^{T}\Sigma_{E}^{-1/2}B_{3}^{*T}G_{1}^{-1/2}G_{1}^{-1/2}B_{3}^{*}\Sigma_{E}^{-1/2}e_{i}=\sum_{i=1}^{I}e_{i}^{T}\Sigma_{E}^{-1/2}B_{3}^{*T}G_{1}^{-1}B_{3}^{*}\Sigma_{E}^{-1/2}e_{i}=tr\left(\Sigma_{E}^{-1/2}B_{3}^{*T}G_{1}^{-1}B_{3}^{*}\Sigma_{E}^{-1/2}\right)=tr\left(B_{2}^{*T}G_{1}^{-1}B_{3}^{*}\Sigma_{E}^{-1}\right).$

### Full conditional for $a_{lE}$

$$\begin{array}{c} f\left(a_{lE}|ELSE\right)\propto f\left(\Sigma_{E}|a_{lE}\right)f\left(a_{lE}\right)\propto \frac{1}{{\left(a_{lE}\right)}^{\frac{\nu_{E}+I}{2}+1}}\exp\left(-\frac{\frac{1}{A_{lE}^{2}}+\nu_{E}{\left(\Sigma_{E}^{-1}\right)}_{ll}}{a_{lE}}\right)\\ \propto IG\left(\frac{\nu_{E}+I}{2},1/A_{lE}^{2}+\nu_{E}{\left(\Sigma_{E}^{-1}\right)}_{ll}\right) \end{array}$$ (B8)

### Full conditional for $\omega_{ijk}^{\left(l\right)}$

$$f\left(\omega_{ijk}^{\left(l\right)}|ELSE\right)∼PG\left(b=y_{ijk}^{\left(l\right)}+r,d=\eta_{ijk}^{*\left(l\right)}\right)$$ (B9)

### Full conditional for $\Sigma_{c}$

$$\begin{array}{c} f\left(\Sigma_{c}|-\right)\propto \frac{1}{{\left|\Sigma_{c}\right|}^{\frac{KJI}{2}}}\exp\left[-\frac{1}{2}c^{T}\left(I_{KJI}⊗\Sigma_{c}{}^{-1}\right)c\right]f\left(\Sigma_{c}\right)\\ \;\propto \frac{1}{{\left|\Sigma_{c}\right|}^{\frac{KJI}{2}}}\exp\left[-\frac{1}{2}c^{T}\left(I_{KJI}⊗\Sigma_{c}^{-1}\right)c\right]\frac{1}{{\left|\Sigma_{c}\right|}^{\frac{v_{c}+2n_{T}}{2}}}\;\exp\left(-\frac{1}{2}tr\left(C_{c}\Sigma_{c}^{-1}\;\right)\right)\propto \\ \;{\left|\Sigma_{c}\right|}^{-\frac{v_{c}+\;KJI+2n_{T}}{2}}\exp\left(-\frac{1}{2}tr\left(C_{c}^{*}\Sigma_{c}^{-1}\;\right)\right)∼IW\left(v_{c}+KJI+n_{T}-1,C_{c}^{*}\right) \end{array}$$ (B10)

where $C_{c}^{*}=C_{c}+B_{4}^{*}B_{4}^{*T},$ where $B_{4}^{*}$ is a matrix with $n_{T}$ rows such that $\mathrm{vec}\left(B_{4}^{*}\right)=c.$

### Full conditional for $a_{lc}$

$$\begin{array}{c} f\left(a_{lc}|ELSE\right)\propto f\left(\Sigma_{c}|a_{lc}\right)f\left(a_{lc}\right)\propto \frac{1}{{\left(a_{lc}\right)}^{\frac{\nu_{c}+I}{2}+1}}\exp\left(-\frac{\frac{1}{A_{lc}^{2}}+\nu_{c}{\left(\Sigma_{c}^{-1}\right)}_{ll}}{a_{lc}}\right)\\ \propto IG\left(\frac{\nu_{c}+I}{2},1/A_{lc}^{2}+\nu_{c}{\left(\Sigma_{c}^{-1}\right)}_{ll}\right) \end{array}$$ (B11)

## References

[bib1] Cavanagh C. R. Chao S. Wang S. Huang B. E. Stephen S., 2013 Genome-wide comparative diversity uncovers multiple targets of selection for improvement in hexaploid wheat landraces and cultivars. Proc. Natl. Acad. Sci. USA 110(20): 8057–8062. 23630259 10.1073/pnas.1217133110 PMC3657823

[bib2] Eddelbuettel D. Sanderson C., 2014 Rcpparmadillo: accelerating R with high-performance C++ linear algebra. Comput. Stat. Data Anal. 71: 1054–1063 10.1016/j.csda.2013.02.005.

[bib3] Harville D. A., 2014 The need for more emphasis on prediction: a “nondenominational” model-based approach. Am. Stat. 68(2): 71–83. 10.1080/00031305.2014.897257 PMC4062083 24954949

[bib4] Henderson C. R. Quaas R. L., 1976 Multiple trait evaluation using relatives’ records. J. Anim. Sci. 43(6): 1188–1197.

[bib5] Jiang J. Zhang Q. Ma L. Li J. Wang Z., 2015 Joint prediction of multiple quantitative traits using a Bayesian multivariate antedependence model. Heredity 115(1): 29–36. 25873147 10.1038/hdy.2015.9 PMC4815501

[bib6] Ma J. Kockelman K. M. Damien P., 2008 A multivariate Poisson-lognormal regression model for prediction of crash counts by severity, using Bayesian methods. Accid. Anal. Prev. 40(3): 964–975. 18460364 10.1016/j.aap.2007.11.002

[bib7] Montesinos-López O. A. Montesinos-López A. Pérez-Rodríguez P. Eskridge K. He X., 2015 Genomic prediction models for count data. J. Agric. Biol. Environ. Stat. 20(4): 533–554.

[bib8] Montesinos-López A. Montesinos-López O. A. Crossa J. Burgueño J. Eskridge K., 2016 a Genomic bayesian prediction model for count data with genotype × environment interaction. G3: 6(5): 1165–1177. 26921298 10.1534/g3.116.028118 PMC4856070

[bib9] Montesinos-López O. A. Montesinos-López A. Crossa J. Toledo F. H. Pérez-Hernández O., 2016 b A genomic Bayesian multi-trait and multi-environment model. G3 6(9):2725–2744. 27342738 10.1534/g3.116.032359 PMC5015931

[bib10] Park T. van Dyk D. A., 2009 Partially collapsed Gibbs samplers: illustrations and applications. J. Comput. Graph. Stat. 18: 283–305.

[bib11] Pollak E. J. Van der Werf J. Quaas R. L., 1984 Selection bias and multiple trait evaluation. J. Dairy Sci. 67(7): 1590–1595.

[bib12] Polson N. G. Scott J. G. Windle J., 2013 Bayesian inference for logistic models using Pólya–Gamma latent variables. J. Am. Stat. Assoc. 108: 1339–1349.

[bib13] R Core Team, 2016 *R: A Language and Environment for Statistical Computing* R Foundation for Statistical Computing, Vienna, Austria. Available at: http://www.R-project.org/. Accessed: January 10, 2017.

[bib14] Schaeffer L. R., 1984 Sire and cow evaluation under multiple trait models. J. Dairy Sci. 67(7): 1567–1580.

[bib15] Williams M. S. Ebel E. D., 2012 Methods for fitting the Poisson-lognormal distribution to microbial testing data. Food Control 27(1): 73–80.

